# Genotoxic Treatment Enhances Immune Response in a Genetic Model of Lung Cancer

**DOI:** 10.3390/cancers13143595

**Published:** 2021-07-18

**Authors:** Pasquale Saggese, Cesar A. Martinez, Linh M. Tran, Raymond Lim, Camelia Dumitras, Tristan Grogan, David Elashoff, Salehi-Rad Ramin, Steven M. Dubinett, Bin Liu, Claudio Scafoglio

**Affiliations:** 1 Division of Pulmonary and Critical Care Medicine, David Geffen School of Medicine, University of California Los Angeles, Los Angeles, CA 90095, USA; psaggese@mednet.ucla.edu (P.S.); cesaramartinez@mednet.ucla.edu (C.A.M.); linhmtran@mednet.ucla.edu (L.M.T.); RaymondLim@mednet.ucla.edu (R.L.); CDumitras@mednet.ucla.edu (C.D.); rsalehirad@mednet.ucla.edu (S.-R.R.); sdubinett@mednet.ucla.edu (S.M.D.); bliu@mednet.ucla.edu (B.L.); 2 Department of Medicine Statistics Core, David Geffen School of Medicine, University of California Los Angeles, Los Angeles, CA 90095, USA; TGrogan@mednet.ucla.edu (T.G.); DElashoff@mednet.ucla.edu (D.E.)

**Keywords:** cancer immunotherapy, murine models, lung cancer

## Abstract

**Simple Summary:**

Immunotherapy has yielded exciting results against lung cancer, but its efficacy is limited to a small percentage of patients, highlighting the necessity to develop new experimental approaches. The currently available models for pre-clinical studies fail to reproduce the biological features of human cancers. Genetically engineered murine models (GEMMs) are driven by key mutations identified in patients, but they do not recapitulate the complex mutational landscape of human cancers, thus failing to activate the immune system appropriately. On the other side, carcinogen-induced models have appropriate mutational burden, but they require much longer experimental times and have inconsistency of results. We developed a hybrid model in which lung tumors are driven by genetically engineered oncogenic mutations in mice, with increased mutational load induced by in vivo treatment with a carcinogen. This model more closely mimics the complexity of human lung cancer and is suitable for pre-clinical immunotherapy studies.

**Abstract:**

Recent advances in immunotherapy have reshaped the clinical management of lung cancer, and immune checkpoint inhibitors (ICIs) are now first-line treatment for advanced lung cancer. However, the majority of patients do not respond to ICIs as single agents, and many develop resistance after initial responses. Therefore, there is urgent need to improve the current ICI strategies. Murine models currently available for pre-clinical studies have serious limitations for evaluating novel immunotherapies. GEMMs are reliable and predictable models driven by oncogenic mutations mirroring those found in cancer patients. However, they lack the mutational burden of human cancers and thus do not elicit proper immune surveillance. Carcinogen-induced models are characterized by mutational burden that more closely resembles human cancer, but they often require extremely long experimental times with inconsistent results. Here, we present a hybrid model in which genetically engineered mice are exposed to the carcinogen *N*-Methyl-*N*-Nitrosourea (MNU) to increase tumor mutational burden (TMB), induce early-stage immune responses, and enhance susceptibility to ICIs. We anticipate that this model will be useful for pre-clinical evaluation of novel immunotherapies.

## 1. Introduction

Recent advances in immunotherapy have revolutionized the treatment of lung cancer [1,2]. ICIs are now first-line treatment for metastatic disease both in non-small cell lung cancer (NSCLC) and in small cell lung cancer, either in monotherapy or in combination with chemotherapy [3]. Recently, their use has been extended to non-metastatic stage III NSCLC after concurrent chemo-radiotherapy [4], and their use has been proposed to treat early-stage disease [5,6]. Despite durable and robust clinical benefit in a subset of patients, the majority of patients do not respond to ICIs, highlighting the necessity for further research to develop effective therapies. GEMMs of cancer are essential for the pre-clinical development of new treatment strategies against cancer. One of the most frequently used GEMMs of lung cancer is the KP model, which is driven by the *Kras*^G12D^ mutation and *Tp53* deletion. *KRAS* and *TP53* are the most frequently mutated genes in NSCLC [7,8]; in this conditional model, the activation of oncogenic *Kras* and the deletion of *Tp53* can be induced in the lungs by inhalation of adenoviral particles expressing Cre recombinase (AdenoCre, Viral Vector Core Facility, University of Iowa, Iowa City, IA, USA). After tumor induction with AdenoCre, KP mice develop multiple lung adenocarcinomas. KP mice are fully immune-competent and have been reported to mount a robust anti-tumor immune response [9,10,11]. However, it has become clear from pre-clinical and clinical studies that a major determinant of anti-tumor immune reaction and response to immunotherapies is the TMB, which in turn determines the load of neoantigens that stimulate the anti-tumor immune responses [12]. Kras-driven GEMMs are not good models to study tumor-specific immune responses and to test novel immunotherapies, because they have a significantly lower TMB than human lung tumors or murine tumors in carcinogen-induced models [13].

One of the widely utilized carcinogen-induced murine models of lung cancer is via the administration of the alkylating agent *N*-methyl-*N*-nitrosourea (MNU). Mice treated with MNU via intraperitoneal (i.p.) injection develop multiple tumors in different organs, with predominance of hematopoietic, gastrointestinal, and lung tumors bearing significantly increased TMB [14]. MNU has been widely used to establish carcinogen-induced lung cancer models [13,15]. One drawback of the carcinogen-induced murine model is the stochastic nature of tumorigenesis with low predictability and long latency of tumor development, limiting the utility of these models in evaluating experimental treatments.

Recently, an in vitro hybrid model has been developed in which cancer cell lines established from lung tumors from *Kras*-driven GEMMs are treated in vitro with MNU. This treatment dramatically increases the TMB to levels similar to those observed in lung cancer patients, and increases the immunogenicity of these tumors when the mutated cells are re-injected in syngeneic mice [16]. This subcutaneous model is suitable for studying novel immunotherapies. However, the cancer cell lines are established from advanced tumors, and cannot be used for studying the early stages of lung cancer development and the interactions of normal, neoplastic, and immune cells in the lung microenvironment.

In order to create a physiologically relevant and reliable model for pre-clinical testing of immunotherapies, we have developed an in vivo hybrid murine model in which lung adenocarcinomas are driven by *Kras* mutation and *Tp53* deletion in the KP GEMMs, and the TMB is increased to levels similar to human cancer by the concomitant administration of MNU in vivo. We show that MNU treatment of KP mice causes sustained T cell infiltration and immune checkpoint activation in the tumor microenvironment, accompanied by a better response to ICI therapy.

## 2. Results

### 2.1. MNU Treatment Increases the Mutation Burden in a Genetically Engineered Murine Model of Lung Cancer

**Figure 1:**
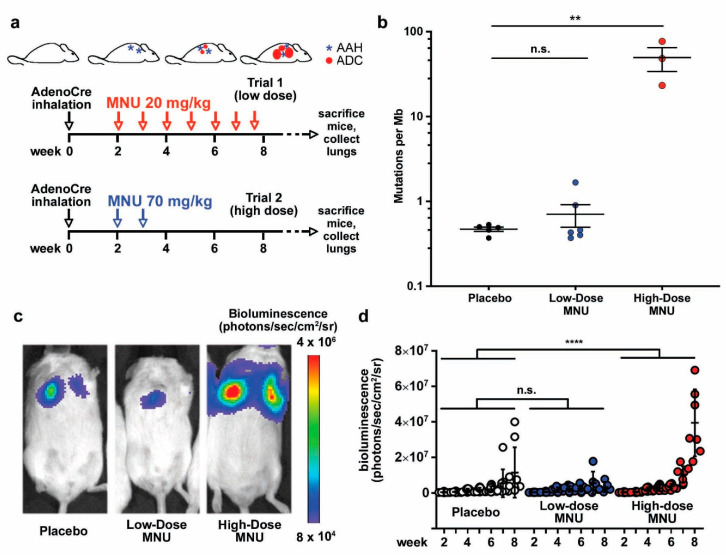
MNU treatment increases the mutation burden and affects tumor growth in a genetically engineered murine model (GEMM) of lung cancer. (**a**) Schematic representation of the treatment regimens. Lung tumors are induced in KP mice by intranasal instillation of AdenoCre. After AdenoCre inhalation, mice develop pre-malignant lesions (atypical adenomatous hyperplasia; AAH) and adenocarcinomas (ADC) with a predictable timing. Mice were treated with either eight low doses (20 mg/kg, trial 1) of MNU or with two high doses (70 mg/kg, trial 2), both on a weekly basis. Each of the two treatment regimens had a corresponding placebo group. At the end of the trials, the mice were euthanized and the tumors collected. (**b**) Mutation burden was measured in tumors from the placebo and the two treatment regimens by WES at the end of the trials, and expressed as mutations per Mb, mean ± standard error of the mean (SEM). The differences in mutation numbers were evaluated by Student’s *t*-test. (**c**,**d**) Tumor burden was estimated in the placebo mice and in the two MNU regimens by weekly BLI. (**c**) Representative images of BLI in the placebo and the two treatment regimens at 8 weeks after tumor induction. (**d**) Quantification of the BLI in the placebo and the two MNU regimens (low-dose and high-dose). The differences in tumor burden (log scale) in the different treatment groups were assessed using a generalized estimating equation (GEE) model [18] to accurately account for the same mouse being measured multiple times. ** *p* < 0.01; **** *p* < 0.0001.

To establish a novel genetically engineered hybrid model to study immunotherapy of lung adenocarcinoma, we used our conditional KP GEMM. Our previous results revealed that tumors start to develop in the murine lungs two weeks after AdenoCre administration in this model [17]. To increase the TMB in the KP tumors, we treated mice with MNU by i.p. injection starting two weeks after AdenoCre administration. We tested two treatment regimens, as detailed in the Materials and Methods section: in Trial 1, the mice received eight weekly MNU doses of 20 mg/kg (“low dose”), whereas in Trial 2, two weekly MNU doses of 70 mg/kg were administered (“high dose”) (Figure 1a). At the end of the trials, the tumors were collected for whole exome sequencing (WES), which revealed no difference in the TMB between the low-dose MNU and the placebo groups (Figure 1b). However, mice treated with high-dose MNU had significantly higher mutational loads compared with their placebo littermates (Figure 1b). These results suggest that low weekly doses of MNU cause a repairable DNA damage, whereas high doses of MNU produce a genotoxic stress that overwhelms the cellular DNA repair mechanisms and/or escapes immune surveillance, leading to accumulation of mutations. Tumor growth was measured by in vivo bioluminescence imaging (BLI) of the luciferase activity derived from the engineered conditional luciferase transgene in the KP model. BLI revealed that the low-dose MNU group did not have significant difference in tumor burden compared with the placebo group, whereas the high-dose MNU cohort had larger tumors than the placebo group, as evidenced by the significantly higher values in bioluminescence (Figure 1c,d).

### 2.2. MNU Causes Sustained Tumor Lymphocyte Infiltration in KP Tumors

To characterize how MNU affects the immune response within our models, we performed time course trials on both the high- and low-dose treatment groups. To estimate changes in anti-tumor immune response induced by MNU, we measured the number of tumor-infiltrating lymphocytes (TILs) by immunohistochemical stain with an anti-CD3 antibody. Mice were treated as detailed in the Materials and Methods section (trials 3 and 4) and were euthanized at week 3 and week 6 timepoints. The lungs were formalin-fixed, paraffin-embedded and stained for CD3.

**Figure 2:**
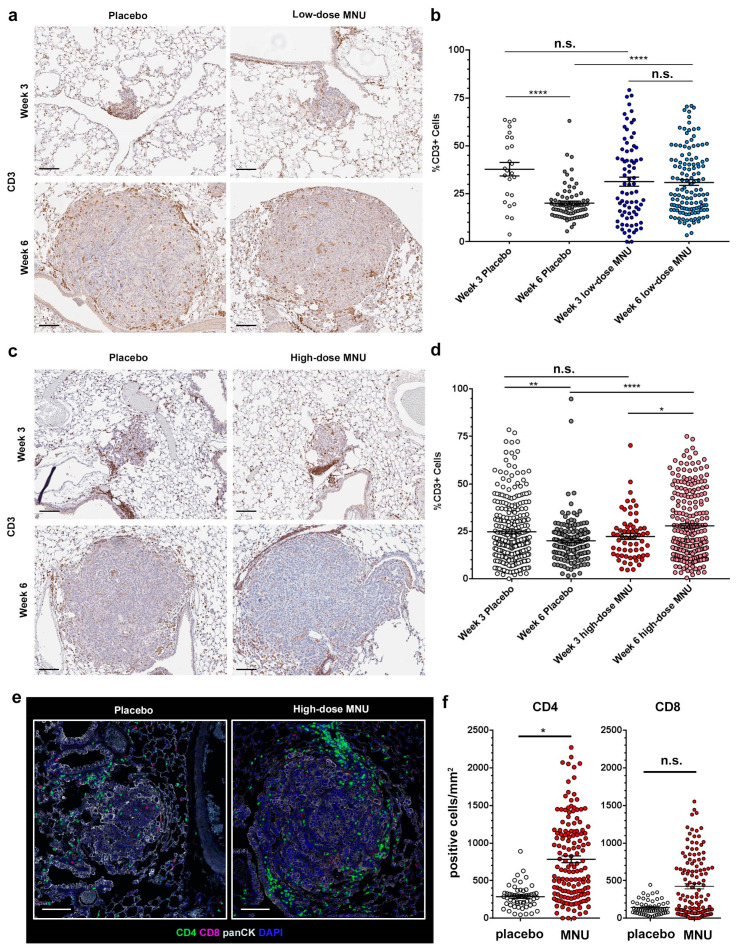
MNU treatment causes sustained lymphocyte infiltration in KP tumors. KP mice were treated with low-dose or high-dose MNU for either 3 or 6 weeks, followed by lung extraction and fixation for immunohistochemistry. At both time points, each MNU regimen had a corresponding placebo group. TIL number was evaluated by immunohistochemistry for CD3. (**a**) Representative pictures of CD3 staining in KP tumors treated with either placebo or low-dose MNU, as indicated. (**b**) Quantification of the number of TILs in each tumor of the placebo and low-MNU group at 3 and 6 weeks, as measured by percentage of CD3-positive cells in the tumor section. The measurement was made with QuPath software by identifying total cells based on the hematoxylin counterstain and then quantifying the DAB signal in each cell. (**c**) Representative pictures of CD3 staining in KP tumors treated with either placebo or high-dose MNU, as indicated. (**d**) Quantification of the number of TILs in each tumor of the placebo and low-MNU group at 3 and 6 weeks, as measured by percentage of CD3-positive cells in the tumor section. The error bars show mean ± SEM. The statistical significance of the differences in TILs in the MNU groups vs. the respective placebo groups was evaluated by Student’s *t*-test. (**e,f**) The lung sections of mice treated with either placebo or high-dose MNU at the 6 weeks time point were stained by multiple immunofluorescence with antibodies for CD4, CD8, pan-cytokeratin (panCK), and counterstained with DAPI, as indicated. (**c**) Representative pictures. (**d**) Quantification of the number of cells with a positive signal for CD4 (left panel) or CD8 (right panel). * *p <* 0.05, ** *p <* 0.01, **** *p <* 0.0001. n.s. not significant. Scar bars: 100 µm.

In the low-dose MNU trial (trial 3), placebo mice had a significant decrease in the percentage of CD3+ cells in the tumor from week 3 to week 6, which dropped from 37.8 ± 3.5% to 20.2 ± 1.0% of the total cells in the tumor microenvironment (Figure 2a). MNU treatment did not cause a significant change in the number of TILs at week 3, with a percentage of CD3+ cells of 31.3 ± 2.4%. However, in the MNU-treated group there was no reduction in TILs as the tumors progressed from week 3 to week 6, with a percentage of CD3+ cells in the tumor at week 6 of 30.9 ± 1.5% (Figure 2a). Whereas at week 3 there was no significant difference in TIL percentage between placebo and MNU treatment, at week 6 the percentage of TILs was significantly higher in the MNU-treated mice than in the control group (*p <* 0.0001).

In the high-dose MNU trial (trial 4), we confirmed that untreated mice had a significant decrease in the percentage of CD3+ cells in tumors between weeks 3 and 6, from 24.8 ± 1.0% to 20.1 ± 1.0% (*p =* 0.0019) (Figure 2b). However, MNU mice had a significant increase in CD3 expression during the same time period, from 22.3 ± 1.5% at week 3 to 27.9 ± 1.2% at week 6 (*p =* 0.019). Whereas at week 3 the difference in TIL number was not significantly different between MNU-treated and placebo mice, at week 6 MNU treatment caused a significant increase in the percentage of CD3+ cells compared with the placebo group (*p <* 0.0001).

These data suggest that both low-dose and high-dose MNU treatment did not increase the initial number of TILs populating the KP tumors but prevented the number of TILs from declining as tumors progressed from week 3 to week 6.

To further characterize the phenotype of the TILs in the MNU treated tumors, we stained the lungs from mice treated with either placebo or high-dose MNU at week 6 with multiple immunofluorescence (MIF). We used a panel including pan-cytokeratin to identify the tumors and CD4/CD8 to characterize the TILs (Figure 2e). Quantification of the signal showed that the number of CD4*^+^* TILs was significantly higher in the MNU group (783 ± 44 cells/mm*^2^*) than in the placebo group (283 ± 21 cells/mm*^2^*), *p =* 0.019 (Figure 2f, left panel). The CD8*^+^* cells showed a trend toward increased number in the MNU-treated group (423 ± 35 cells/mm^2^) than in the placebo group (142 ± 13 cells/mm^2^*)*, but this difference was not statistically significant (*p =* 0.935) (Figure 2f, right panel). These data show that at this time point MNU treatment causes increased number of T helper cells in the TME.

### 2.3. MNU Causes Sustained Immune Checkpoint Activation in KP Tumors

To evaluate the activation of immune checkpoint in the MNU-treated KP mice, we performed immunohistochemistry with an anti-PD-L1 antibody both in the low-dose and the high-dose groups. Cancer cells and antigen-presenting cells upregulate PD-L1 in response to IFN-γ production in the tumor microenvironment. Expression of PD-L1 in tumor cells and in antigen-presenting cells correlates with the efficacy of checkpoint inhibitors in the clinical setting [19].

**Figure 3:**
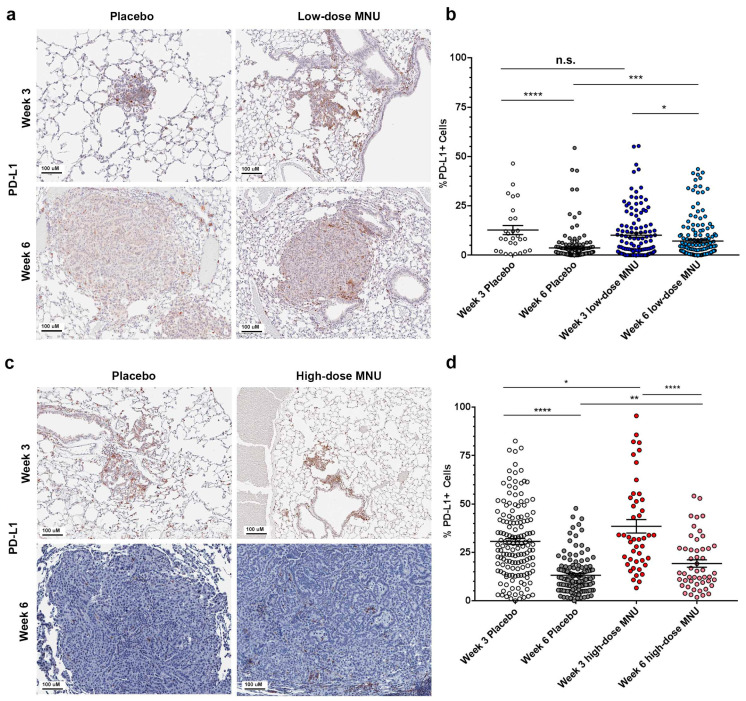
MNU treatment causes sustained immune checkpoint activation in KP tumors. KP mice were treated with low-dose or high-dose MNU for either 3 or 6 weeks, followed by lung extraction and fixation for immunohistochemistry. At both time points, each MNU regimen had a corresponding placebo group. Immune checkpoint activation was evaluated by immunohistochemistry for PD-L1 in tumors. (**a**) Representative pictures of PD-L1 staining KP tumors treated with either placebo or low-dose MNU, as indicated. (**b**) Quantification of the immune checkpoint activation in each tumor of the placebo and low-MNU group at 3 and 6 weeks, as measured by percentage of PD-L1-positive cells in the tumor section. (**c**) Representative pictures of immune checkpoint activation in KP tumors treated with either placebo or high-dose MNU, as indicated. (**d**) Quantification of the immune checkpoint activation in each tumor of the placebo and low-MNU group at 3 and 6 weeks, as measured by percentage of PD-L1-positive cells in the tumor section. The statistical significance of the differences in PD-L1 expression in the MNU groups vs. the respective placebo groups was evaluated by Student’s *t*-test. * *p <* 0.05, ** *p <* 0.01, *** *p <* 0.001. **** *p <* 0.0001. n.s. not significant.

In the low-dose MNU trial (trial 3), PD-L1 expression decreased in the placebo mice from week 3 to week 6, from an average of 12.8 ± 2.3% PD-L1-positive cells to 3.7 ± 0.6% (*p <* 0.0001, Figure 3a,b). MNU treatment did not significantly change the percentage of PD-L1+ cells at week 3 (10.2 ± 1.1% in the MNU group vs. 12.8 ± 2.3% in the placebo group), but caused a significant increase of PD-L1 expression at week 6, from 3.7 ± 0.6% to 7.2 ± 0.8% (*p <* 0.001).

In the high-dose MNU trial (trial 4), PD-L1 expression was significantly decreased in the placebo mice from week 3 to week 6, from a mean percentage of PD-L1-positive cells of 30.6 ± 1.5% at week 3 to 13.2 ± 0.9% at week 6 (Figure 3c,d). MNU treatment caused a significant increase in PD-L1 expression at week 3, with 38.4 ± 3.4% positive cells in the treatment vs. 30.6 ± 1.5% in the placebo group (*p =* 0.02), and at week 6, with 19.2 ± 1.9% in the MNU-treated group vs. 13.2 ± 0.9% in the placebo group (*p =* 0.001).

These results showed that the treatment with MNU at low doses did not cause increased PD-L1 expression but attenuated the decline in PD-L1 expression as the tumors progress from week 3 to week 6. The high MNU doses caused a significant increase in PD-L1 expression and attenuated the decline in PD-L1 expression from week 3 and week 6. Taken together, these results suggest that the MNU treatment, by increasing the TMB and TILs in tumors, caused a sustained activation of the PD-L1 immune checkpoint. Low-dose MNU only increased PD-L1 at week 6, whereas high-dose MNU caused a significant increase in PD-L1 both at week 3 and at week 6.

### 2.4. High-Dose MNU Treatment Improves the Response of KP Tumors to Checkpoint Inhibitors

**Figure 4:**
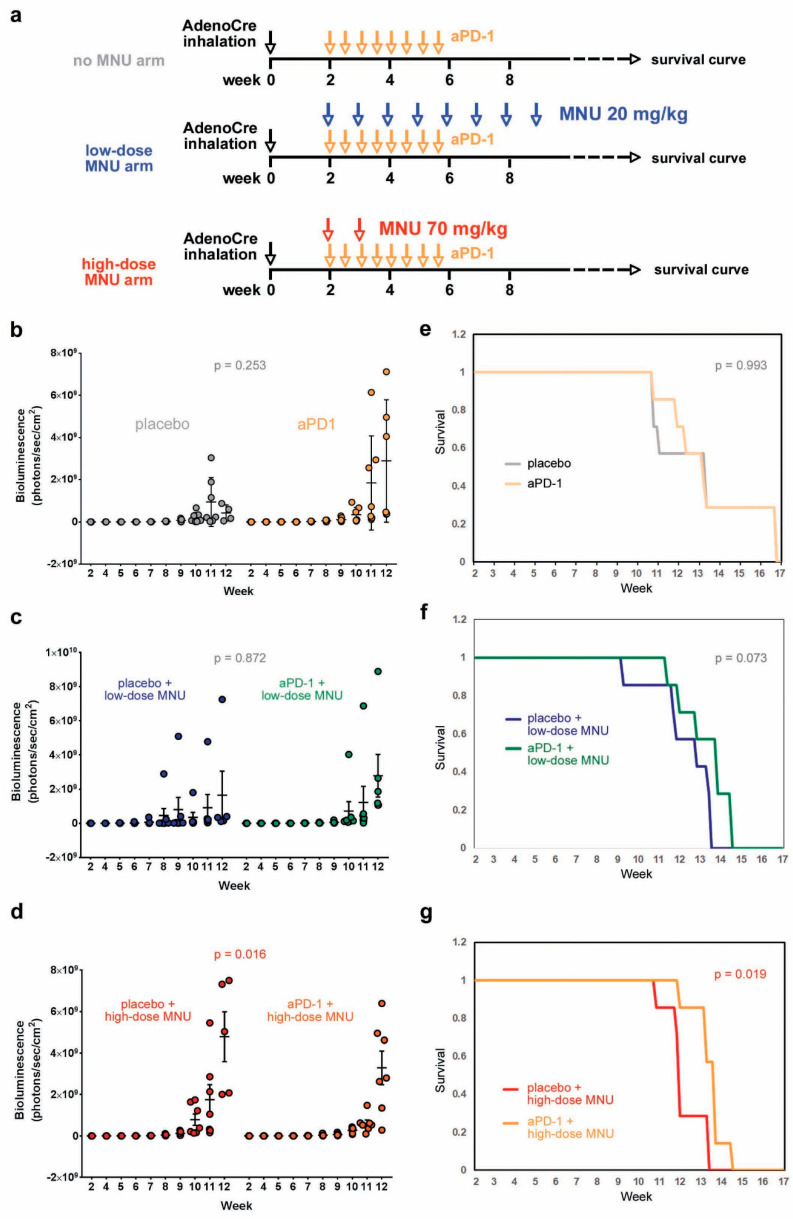
MNU treatment improves the response of KP tumors to anti-PD-1 treatment. (**a**) Tumors were induced in KP mice by AdenoCre inhalation. Two weeks after tumor induction, the mice were treated with either placebo or anti-PD-1 in the absence or presence of two regimens of MNU (low-dose or high-dose). (**a**,**b**) Treatment of KP mice with aPD-1 without MNU exposure. (**b**–**d**) Tumor burden measurements at different time points, as indicated: no-MNU arm (**b**), low-dose MNU arm (**c**), and high-MNU arm (**d**). (**e**–**g**) Survival curves constructed with the Kaplan–Meier method: no-MNU arm (**e**), low-dose MNU arm (**f**), and high-MNU arm (**g**). The statistical significance was evaluated for the tumor burden data with a GEE model, for the survival curves with the log-rank test.

KP mice have been reported to be insensitive to therapies targeting the PD-1/PD-L1 axis [10,11]. Since we observed that MNU exposure caused a sustained activation of the PD-L1 immune checkpoint in KP mice, and that the high MNU doses were more effective than the low doses in increasing PD-L1 expression in the tumors, we next decided to test if MNU treatment sensitizes KP tumors to immune checkpoint inhibitors. We performed a therapeutic trial with an anti-PD1 antibody (8 bi-weekly i.p. doses of 200 mg/mouse) in the KP mice with or without co-treatment with MNU, in two different regimens: low-dose MNU (8 weekly doses of 20 mg/kg) and high-dose MNU (2 weekly doses of 70 mg/kg) (Figure 4a). The tumor burden was evaluated by BLI, and mice were followed after the end of the treatment for up to 4 months, to evaluate the effect on survival.

Anti-PD1 treatment did not cause a significant change in tumor burden in mice with no MNU treatment (Figure 4b) nor in mice treated with low-MNU doses (Figure 4c). However, the high-MNU treatment arm showed a significant reduction in tumor burden between placebo and anti-PD1 treatment groups (Figure 4d). This result is consistent with the previous observation that the high-dose, but not the low-dose MNU treatment, causes an increase in the TMB and PD-L1 expression in KP tumors.

Anti-PD-1 treatment did not change the survival curve in the no-MNU treatment arm (median survival: 9.9 vs. 11.1 weeks in placebo vs. treatment group, Figure 4e). In the low-dose MNU treatment arm, anti-PD1 treatment caused a trend toward increased survival (12.6 weeks vs. 11.6 weeks in the placebo group), which was not statistically significant (*p =* 0.07, Figure 4f). In the high-dose MNU arm, anti-PD1 treatment caused a significant prolongation of survival (median 12.6 weeks) compared with the placebo group (median 10.71 weeks, *p =* 0.019, Figure 4g).

Taken together, these results show that high-dose MNU treatment increases the number of TILs in KP tumors, causes an increased and sustained expression of PD-L1 in the tumor microenvironment, and improves the response to anti-PD1 checkpoint inhibitor.

## 3. Discussion

In this study, we present a novel in vivo hybrid murine model for pre-clinical immunotherapy studies. In this model, lung tumors are induced in genetically engineered mice by the conditional activation of mutant *Kras*^G12D^ and deletion of *Tp53* through intranasal administration of adenoviral vectors expressing the Cre recombinase. After tumor induction, mice are treated with the mutagen MNU to increase the mutational burden, resulting in increased tumor neoantigen load and enhanced anti-tumor immune responses. We show that treatment with MNU causes sustained lymphocyte infiltration and PD-L1 expression in the KP tumors, thus improving the response of these tumors to anti-PD-1 treatment.

This model presents several advantages over previous murine models to study immunotherapy: (1) tumors are reliably driven by *Kras* mutation and *Tp53* deletion, which are the most common driver genetic alterations identified in human lung adenocarcinomas; (2) the MNU treatment significantly increases the TMB, overcoming a limitation posed by the poor immunogenicity of conventional GEMMs; (3) tumorigenesis occurs in the physiologically relevant lung microenvironment; (4) tumors develop in the genetically engineered murine lungs, making this model suitable for studying the earliest stages of lung cancer starting from the pre-malignant phase. Although the current clinical use of immunotherapy is largely limited to advanced disease, several studies are investigating the possibility of using immunotherapy for early-stage lung cancer [2,20].

Even though *KRAS* and *TP53* are commonly mutated in lung adenocarcinoma, several co-existing mutations can modulate anti-cancer immune responses and sensitivity to immunotherapies. *TP53* has been associated with response to ICI independent of other mutations [21], confirming the relevance of our model. However, *STK11* and *KEAP1* mutations have been associated with poor response to immunotherapy [22,23]. In the future, it will be important to extend our in vivo hybrid model to different patterns of co-occurring mutations to study mechanisms of resistance and test novel treatments that can improve response to immunotherapy.

Since the MNU doses used in carcinogen-induced cancer models vary widely in the literature, we tested two different conditions of weekly treatments: two high doses vs. eight low doses. The cumulative doses were similar in these two arms, and both treatment regimens led to significantly sustained TILs and PD-L1 expression in the tumors. However, only the high MNU dose causes a significantly increased TMB and improved response to anti-PD-1 treatment. Since the low MNU dose still causes a sustained T cell infiltration and PD-L1 activation in the TME, we assume that the low dose is sufficient to induce mutations in the lung tumors, but this gradual mutational burden is effectively cleared by the DNA repair systems of cells as well as by the immune system. Consistently, low-dose MNU treatment did not cause a significant reduction of the tumor burden, whereas the mutational burden caused by high-dose MNU overwhelms the endogenous DNA repair system and/or the immune system, resulting in increased tumor burden and a massive increase in the TMB. In an in vivo setting, it is not easy to discern between “on-target” effects on the tumor cells and “off-target” effects on other cells, including the immune system [24]. Cytotoxic agents can enhance anti-cancer immune responses through several mechanisms, including increased neoantigen expression, activation of the innate immune system, and adjuvant effect of cancer cells [24]. According to the dose, cytotoxic agents can either stimulate or suppress the immune responses against cancer [24].

Even if the exact mechanism of increased immunogenicity remains to be determined, the high-dose MNU treatment improves the response of the tumors to anti-PD-1 treatment, suggesting that the high-dose MNU model is suitable for future pre-clinical studies on novel immunotherapies or immune checkpoint inhibitor-based combination treatments.

## 4. Materials and Methods

Mouse model. All animal experiments were approved by the University of California Los Angeles (UCLA) Institutional Animal Care and Use Committee and were performed following the guidelines of the Department of Laboratory Animal Medicine at UCLA. The *Lox-Stop-Lox Kras^G12D^*, *p53^lox/lox^*, *Rosa26-Lox-Stop-Lox-Luc* were kindly provided by Dr. David B. Shackelford (Division of Pulmonary and Critical Care Medicine, David Geffen School of Medicine, UCLA) and bred in our facilities on an FVB background. Lung tumors were induced by intranasal administration of AdenoCre (purchased from the Viral Vector Core Facility, University of Iowa) as previously described [17]. Two weeks after tumor induction, mice were enrolled in different trials, as described below. The tumor burden was evaluated by weekly bioluminescence performed on an IVI Spectrum in vivo imaging system (Perkin Elmer, Waltham, MA, USA) 10′ after i.p. injection of luciferin (150 mg/kg).

Mouse trials. For all mouse trials, mice received the first BLI measurement two weeks post-adenoCre administration for measurement of the baseline tumor burden. Mice were assigned to therapeutic groups so that there were no significant differences in baseline tumor burden, age, sex, or weight among the different groups. MNU (Chemservice, West Chester, PA, USA) was dissolved fresh in sterile saline solution (0.9% NaCl) with 0.05% acetic acid and injected i.p. in the mice soon after preparation of the solution. The groups are described as follows.

Trial 1 (low-dose MNU). Two groups: placebo (*n* = 7) and low-dose (20 mg/kg/week, eight total doses) MNU (*n* = 4). Mice were treated for eight weeks and BLI was measured weekly, followed by sacrifice of all mice by week 10 post-AdenoCre. Some of the tumors (placebo: *n* = 4; low-dose MNU: *n* = 6) were flash-frozen for measuring the tumor burden by whole exome sequencing.

Trial 2 (high-dose MNU). Two groups: placebo (*n* = 4) and high-dose (70 mg/kg/week, two total doses) MNU (*n* = 5). The tumor burden was monitored until week 9 post-AdenoCre, followed by sacrifice of the mice. Some of the tumors (high-dose MNU: *n* = 3) were flash-frozen for measuring the tumor burden by whole exome sequencing.

Trial 1 and trial 2 were repeated in a third experiment with three groups (*n* = 8): placebo, low-dose MNU, and high-dose MNU.

Trial 3 (low-dose MNU, time course). Two arms: 3 week (sacrificed after 3 weekly doses) and 6 week (sacrificed after 6 weekly doses). Each arm had two groups (placebo and low-dose MNU). The weekly dose of MNU was 20 mg/kg. The number of mice for each group is the following: 3 week placebo: *n* = 3; 3 week MNU: *n* = 6; 6 week placebo: *n* = 4; 6 week MNU: *n* = 6. After sacrifice, the lungs were extracted, fixed in formalin and embedded in paraffin for immunohistochemistry. The experiment was repeated twice.

Trial 4 (high-dose MNU, time course). Two arms: 3 week and 6 week. Each arm had two groups (placebo and high-dose MNU). All animals in the high-dose MNU groups received two MNU doses of 70 mg/kg at weeks 2 and 3, followed by sacrifice at either week 3 (one day after the second dose) or at week 6. The number of mice for each group is the following: 3 week placebo: *n* = 8; 3 week MNU: *n* = 8; 6 week placebo: *n* = 8; 6 week MNU: *n* = 9. After sacrifice, the lungs were extracted, fixed in formalin and embedded in paraffin for immunohistochemistry. The experiment was repeated twice.

Trial 5 (combination treatment). Three arms: no MNU, low-MNU (20 mg/kg/week, 8 doses), and high-MNU (70 mg/kg/week, 2 doses). Each arm had two groups: placebo and anti-PD-1 (BioXcell, Lebanon, NH, USA, #BE0146, 200 µg/mouse i.p. injection q3d, 8 total doses). BLI was measured weekly and after all treatments were completed mice were observed until death for survival curve. The experiment was repeated twice.

Statistical analysis. The differences in tumor burden (log scale) in the different treatment groups were assessed using a generalized estimating equation (GEE) model [18] to accurately account for the same mouse being measured multiple times. GEE models were run using SAS V9.4 (Cary, NC, USA) and *p*-values < 0.05 were considered statistically significant.

For the survival analysis, within each MNU group (none, low, high), overall survival was displayed using curves constructed using the Kaplan–Meier method and formally compared between groups (anti-PD1 vs. control) using the log-rank test. Plots and survival analyses were run using IBM SPSS V27 (Armonk, NY, USA) and *p*-values < 0.05 were considered statistically significant.

Tumor mutation burden. Genomic DNA isolation, library preparation, and sequencing. Genomic DNA was extracted from tumors (Qiagen, Germantown, MD, USA, DNeasy blood and tissue kit) for whole exome sequence (WES). Tail DNA from two mice was included as a normal reference for variant calls. Libraries for WES were prepared using the Kapa Hyper Prep Kit (Roche, Basel, Switzerland, KK8504) followed by exome enrichment with SeqCap EZ Share Developer Probe (Roche, 08333025001). Sequencing was performed on Hiseq3000 instrument as 150 bp pair-end runs with the aim of 100× depth at UCLA TCGB Core facility.

WES preprocessing. Sequence reads were aligned to the mouse genome *(mm10*) with Burrows-Wheeler Aligner (v 0.7.17), then marked for duplicates, indel realignment, and re-calibrated by Genome Analysis Toolkit (GATK v 3.8.0).

Mutation Calling and Annotation. Strelka2 was utilized with default parameters for variant calls between tumor and the individual normal tail genome. Variants called based on both reference genomes were subjected to further down-stream analyses. Finally, a variant was called a somatic mutation if (1) it was not in the germline mutation panel, determined from the FVB and 129-E tail genomes, (2) it was not supported by any read in the associated normal genome, (3) it was detected by at least 5 reads in cell lines, and (4) its variant allelic frequency (VAF) was > 0.1. The variants that passed these criteria were then annotated by Ensembl Variant Effect Predictor (European Molecular Biology Laboratory’s European Bioinformatics Institute, Hinxton, UK) as nonsynonymous mutations based on Ensembl gene annotation (version 93).

Immunohistochemistry (IHC). Lungs from the mice in different trials were sliced into 4 µm sections and used for IHC as described before [17,25]. Briefly, slides were de-paraffinized by overnight incubation at 65 °C, rehydrated by serial passages in xylenes and decreasing concentrations of ethanol, boiled for 20′ in 10 mM citrate buffer (pH 6.0) for antigen retrieval, blocked in 5% goat serum for 1h at room temperature, incubated overnight with primary antibodies at 4 °C and for 1h at room temperature with biotin-labeled secondary antibodies (Vector Labs, Burlingame, CA, USA), followed by incubation with avidin-biotin peroxidase complex (ABC, Vector Labs) for 1h and ImmPACT DAB (Vector Labs) for 1′. Counterstain was performed with Harris’ hematoxylin diluted 1:5 in water. The following antibodies were used: CD3 (rabbit polyclonal, Dako #A0452, 1/2000 dilution, Agilent Technologies, Santa Clara, CA, USA), PD-L1 (rabbit polyclonal, Cell Signaling #64988, 1/200 dilution). The immunohistochemistry signal was quantified by semi-quantitative assessment of percentage of positive cells by QuPath analysis (University of Edonburg, Scotland, UK) [26]. In lung sections, regions of interest were drawn to encompass single tumors. Within the tumors, the total number of cells was evaluated based on the hematoxylin counterstain with the “cell detection” function of QuPath. The percentage of CD3 or PD-L1 positive cells was evaluated by the intensity of DAB staining in cells. The differences between treatment groups were analyzed by Student’s *t*-test.

Multiple immunofluorescence (MIF). MIF was performed utilizing the Ventana Discovery Ultra (Roche) and Opal fluorophores (Akoya Biosciences). Five micrometer thick tissue sections on Superfrost microscopic slides (VWR International) were deparaffinized using EZ-Prep reagent (Roche) followed by antigen retrieval in CC1 buffer (pH 9, 95 °C; Roche). Discovery Inhibitor (Roche) was applied to inhibit enzymatic activities followed by 3 sequential rounds of staining. Each round included the addition of a primary antibody followed by detection using the OmniMap secondary antibody (Roche). Signal amplification was performed utilizing Opal fluorophores in the conditions suggested by the manufacturer. Between rounds of staining the tissue sections underwent heat-induced epitope retrieval to remove the primary-secondary-HRP antibody complexes before staining with the subsequent antibody. The primary antibodies and corresponding fluorophores are PanCK (DAKO) in Opal 480; CD3 (Roche) in Opal 520; CD8 (Cell Signaling) in Opal 690. The slides were then counterstained with Spectral DAPI (Akoya Biosciences) and mounted with ProLong Diamond antifade mounting medium (Thermo Fisher Scientific). Stained slides were imaged using the Vectra Polaris imaging system (Akoya Biosciences, Marlborough, MA, USA). A whole slide scan was acquired with 20× resolution. Following image capture, regions of interest (ROIs) were selected on each slide using the Phenochart viewer (Akoya Biosciences) and imported into the inForm software 2.4.4 (Akoya Biosciences) followed by unmixing the spectral libraries, cell segmentation and cell phenotyping. ROIs corresponding to whole tumor regions from each slide were then analyzed to identify and characterize the cells. The representative images were exported using inForm software 2.4.4 following spectral unmixing. CD4 and CD8 expression values/counts were log-transformed (natural log +1) prior to analysis to better meet statistical assumptions (e.g., normality, heteroscedasticity) and formally compared between groups using the GEE modeling framework with a repeated mouse effect (multiple tumors per mouse) with the compound symmetry covariance structure. Analysis was run using SAS V9.4 (SAS Institute, Cary, NC) and *p*-values <0.05 were considered statistically significant.

## 5. Conclusions

Here, we present a hybrid model in which lung tumors are induced by conditional activation of transgenic *Kras*^G12D^ mutant and deletion of *Tp53* in the murine lungs, and a short-time treatment with two weekly doses of carcinogen MNU induce increased TMB, lymphocyte infiltration and PD-L1 expression in tumors, and improved response to anti-PD-1 treatment. We anticipate that this model will be valuable for pre-clinical evaluation of novel immunotherapies.

## Data Availability

The data presented in this study are available on request from the corresponding author.
